# Development of Spontaneous Echocardiographic Contrast After Transarterial Occlusion of a Patent Ductus Arteriosus in an Adult Dog With Concurrent Pulmonary Hypertension

**DOI:** 10.3389/fvets.2020.00103

**Published:** 2020-02-26

**Authors:** Randolph L. Winter, Julia D. Remaks, Daniel K. Newhard

**Affiliations:** ^1^Department of Veterinary Clinical Sciences, College of Veterinary Medicine, The Ohio State University, Columbus, OH, United States; ^2^Department of Small Animal Clinical Sciences, College of Veterinary Medicine and Biomedical Sciences, Auburn University, Auburn, AL, United States

**Keywords:** canine, congenital, echocardiography, interventional, smoke

## Abstract

An 8-year-old intact female Chihuahua was presented for evaluation and possible occlusion of a previously diagnosed patent ductus arteriosus (PDA). Transthoracic echocardiography revealed left ventricular and left atrial enlargement, enlargement of the main pulmonary artery, and a PDA with bidirectional shunting. Tricuspid regurgitant velocities suggested moderate pulmonary hypertension. The PDA was occluded with an Amplatz® Canine Duct Occluder using a transarterial approach on the following day. No immediate complications were observed other than an acute decrease in left ventricular systolic function. One day after the PDA occlusion transthoracic echocardiography revealed no residual ductal flow, but there was spontaneous echocardiographic contrast in the left ventricle. The patient was discharged with sildenafil, pimobendan, and clopidogrel. Five weeks later when the patient was presented for a recheck examination, the previously documented spontaneous echocardiographic contrast was no longer present. Finding spontaneous echocardiographic contrast in the dog has not previously been reported in association with PDA occlusion.

## Background

Patent ductus arteriosus (PDA) is a common congenital defect in the dog (1). A PDA represents a persistence patency of a fetal structure which connects the descending aorta to the main pulmonary artery. Although useful for systemic oxygenation in the fetal circulation, the PDA should spontaneously close shortly after an animal takes its first breath. The natural consequences of this lesion include left heart volume overload with or without progression to cardiogenic pulmonary edema, pulmonary hypertension, and death (2, 3).

Surgical ligation or device-based occlusion of the PDA have been employed historically for dogs, and occlusion with an Amplatz® Canine Duct Occluder (ACDO) being commonplace today (3–5). Occlusion of a PDA with an ACDO is a minimally-invasive procedure. Complications of utilizing an ACDO are rare but reported to include acute embolization (5), delayed embolization (6), and device-associated endocarditis (7).

## Case Presentation

Written informed consent was obtained from the owner for the publication of this case report. An 8-year-old, intact, female Chihuahua weighing 2.8 kg was referred to the Auburn University Veterinary Teaching Hospital for evaluation and possible occlusion of a PDA. The dog had been presented to a local emergency clinic several days prior for increased respiratory effort, lethargy, and coughing. Thoracic radiography and transthoracic echocardiography were performed, and a PDA, pulmonary hypertension (PH), and possible cardiogenic pulmonary edema were diagnosed at the emergency clinic. The patient was kept in a supplemental oxygen cage overnight, and the following medications were started: furosemide (1.8 mg/kg orally twice daily); theophylline (8.9 mg/kg orally twice daily); and sildenafil (1.8 mg/kg orally twice daily). Improvements in respiratory rate and frequency of coughing were observed, so the dog was discharged the following morning. Occlusion of the PDA was recommended.

On examination at Auburn University, the dog was quiet and alert, rectal temperature was 37.6°C, heart rate was 132 beats/minute, and the respiratory rate was 48 breaths/minute. Femoral arterial pulse strength was bounding. Normal bronchovesicular sounds were auscultated bilaterally. Cardiac auscultation revealed a grade 3/6 left basilar continuous murmur. A prominent cardiac impulse was palpated at the right hemithorax, equal in strength to that of the left hemithorax.

**Figure 1:**
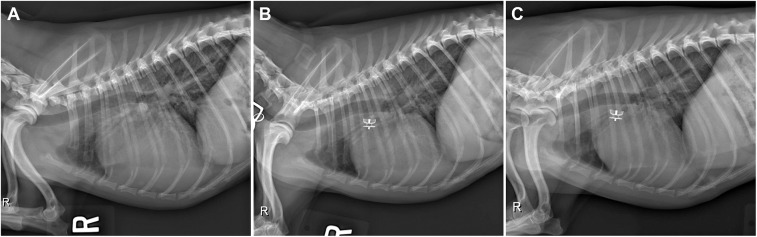
**(A)** Pre-procedural right lateral radiograph demonstrating biventricular enlargement, pulmonary overcirculation, and enlargement of the ascending aorta. **(B)** Right lateral radiograph obtained ~24 h after ACDO deployment. Note the decrease in pulmonary overcirculation and the prominent ascending aorta. **(C)** Right lateral radiograph obtained ~5 weeks after ACDO deployment. Note the resolution of pulmonary overcirculation, decrease in heart size compared to the pre-procedural radiograph, and the unchanged position of the ACDO. ACDO, Amplatz® Canine Duct Occluder.

Thoracic radiographs (Figure 1A) revealed moderate enlargement of the left ventricle (LV) with dorsal displacement of the trachea, mild enlargement and rounding of the right side of the heart, mild enlargement of the left atrium (LA), and a vertebral heart size of 12.6 (normal ≤10.5) (8). The main pulmonary artery (MPA) was enlarged, and there was a focal dilation of the proximal descending aorta (Ao). Pulmonary overcirculation and enlarged peripheral pulmonary arteries were observed, but there was no evidence of active pulmonary edema. The pulmonary parenchyma was considered normal.

**Figure 2:**
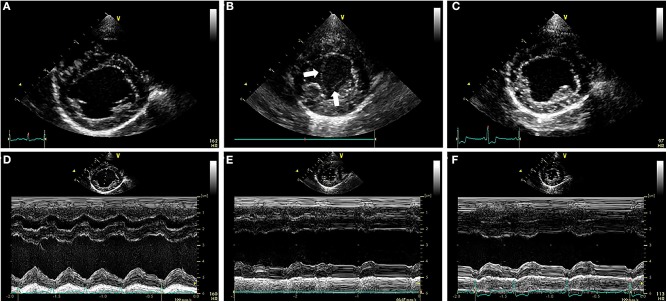
**(A–C)** Right parasternal short axis images of the ventricles during the pre-procedural TTE exam **(A)**, the TTE exam performed ~24 h after ACDO deployment **(B)**, and the TTE exam performed ~5 weeks after ACDO deployment **(C)**. **(D–F)** M-mode images obtained at the times that the images in **(A–C)** were recorded, with each M-mode image directly below its corresponding 2D image above in **(A–C)**. Note the SEC (white arrows) present in the LV at the TTE exam performed ~24 h after ACDO deployment **(B)**. Also note the decrease in FS% present 24 h after ACDO deployment **(E)** compared to the pre-procedural TTE exam **(D)** which has improved by the TTE exam ~5 weeks after ACDO deployment **(F)**. ACDO, Amplatz® Canine Duct Occluder; FS%, fractional shortening percentage; LV, left ventricle; SEC, spontaneous echocardiographic contrast; TTE, transthoracic echocardiography.

**Figure 3:**
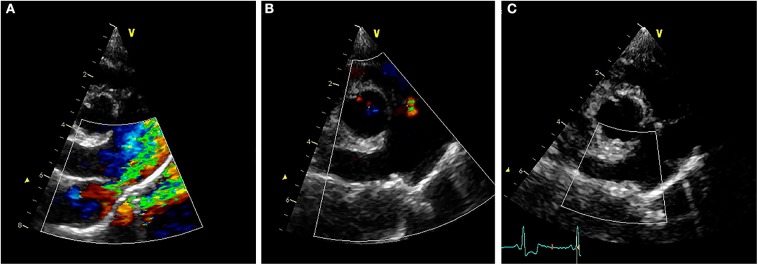
**(A)** Pre-procedural right parasternal short axis TTE view demonstrating severe enlargement of the MPA and RPA, with turbulent blood shunting through the PDA into the MPA during systole. **(B)** Right parasternal short axis TTE view obtained ~24 h after ACDO deployment, demonstrating pulmonic insufficiency and an appropriately positioned ACDO with no residual ductal flow. **(C)** Right parasternal short axis TTE view obtained ~5 weeks after ACDO deployment, demonstrating an unchanged ACDO position and lack of residual ductal flow. ACDO, Amplatz® Canine Duct Occluder; MPA, main pulmonary artery; PDA, patent ductus arteriosus; RPA, right pulmonary artery; TTE, transthoracic echocardiography.

Transthoracic echocardiography (TTE) revealed (Figures 2A,D) LV internal diameters normalized to body weight which were enlarged at end-diastole and normal at end-systole (1.92 and 0.97, respectively) (9). The LA was mildly enlarged based on long-axis 2D measurements, and the LA to aortic root ratio was 2.9 (10). Mitral valve leaflets were mildly thickened and allowed a mild amount of regurgitation. The MPA was severely enlarged with a diameter of (2.0 cm), which was in excess of the aortic root diameter (1.6 cm). The right ventricle (RV) was mildly dilated and had moderate concentric hypertrophy. A PDA (Figure 3A) with bidirectional shunting of blood was observed, with a peak left-to-right systolic pressure gradient of 64 mmHg. The right atrium was normal in size, and there was a mild amount of tricuspid regurgitation. The maximal tricuspid regurgitant velocity (4.13 m/s, estimated RV to RA pressure gradient of 68 mmHg) as well as the changes to the MPA and right heart supported at least moderate PH (11).

Further diagnostics included a serum biochemistry panel, hematocrit, and total serum solids. The hematocrit was 46% (normal 40–59%), and the total serum solids concentration was 6.0 g/dL (normal 5.0–8.0 g/dL). Serum potassium concentration was mildly low at 3.4 mmol/L (normal 3.6–4.9 mmol/L), and this was attributed to the recent furosemide use.

**Figure 4:**
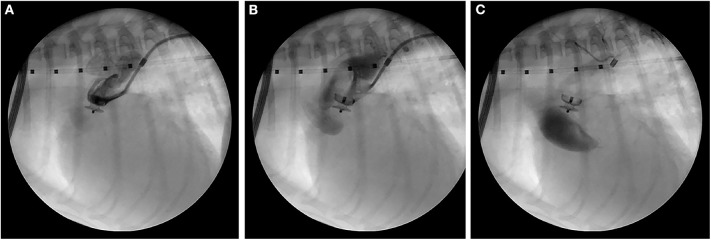
Right lateral fluoroscopic images of the final selective angiogram performed ~5 min after ACDO deployment near the beginning of the contrast injection **(A)**, near the end of the contrast injection **(B)**, and 10 heart beats after the completion of the contrast injection **(C)**. ACDO, Amplatz® Canine Duct Occluder.

Anesthesia was induced on the following day, and the right femoral artery was exposed via a surgical cutdown. Access to the right femoral artery was obtained using the modified Seldinger technique, and a 0.018” x 150 cm hydrophilic guidewire^1^ was advanced into the descending aorta using fluoroscopic guidance. A 4Fr guiding sheath^2^ was advanced over the guidewire into the descending thoracic aorta. Selective angiography confirmed a primarily left-to-right shunting PDA with an ampulla diameter of 4.1 mm and a minimal ductal diameter of 2.1 mm. A 4 mm ACDO^3^ was successfully placed across the pulmonary ostium. Selective angiography revealed mild residual shunting, and gentle manipulation of the ACDO failed to dislodge the device. The ACDO was deployed, and the following changes in anesthetic monitoring parameters were observed: an increase in diastolic blood pressure (20 mmHg); and a decrease in heart rate by 40 beats/min. A final selective angiogram (Figure 4) performed ~5 min after the ACDO was deployed revealed trivial residual flow through the PDA, but contrast was prominently noted within the ascending Ao and aortic arch. This contrast in the ascending Ao and aortic root remained even ~10 heart beats after the completion of the injection. The explanation for this was presumed to be an acute decrease in LV stroke volume, however the patient was stable so no specific therapy was instituted. The 4Fr guiding sheath was removed, the femoral artery ligated, and the patient recovered uneventfully from anesthesia.

Diagnostics performed on the following day included a serum biochemistry panel, hematocrit, serum total solids measurement, thoracic radiographs, and transthoracic echocardiography. The patient was azotemic with a blood urea nitrogen concentration of 20.64 mmol/L (normal 3.2–12.14 mmol/L) and a creatinine concentration of 176.8 μmol/L (normal 44.2–141.4 μmol/L). The patient had also been inappetant overnight and was dehydrated (serum total solids 81.0 g/L). Thoracic radiography revealed an appropriate position of the ACDO (Figure 1B), a decrease in heart size to a vertebral heart size of 11.2, and resolution of pulmonary overcirculation. Transthoracic echocardiography revealed a decrease in diameter of the main pulmonary artery (1.8 cm) and a decrease in left atrial size (LA/Ao 2.1) (10). Left ventricular internal dimensions were slightly decreased in diastole compared to the pre-operative exam, with diameters at end-diastole normalized to body weight of 1.84 (9). However, the diameter at end-systole normalized to body weight had increased and was 1.23, and there was a decreased fractional shortening of 18% (Figure 2E). The ACDO was well-visualized, and there was no residual ductal flow (Figure 3B). Pulmonic insufficiency maximal velocity was 2.43 m/s. A mild amount of aortic insufficiency was present. Spontaneous echo contrast was observed in the LV (Figure 2B and Supplemental Video 1) spanning from the peripheral to the mid-portion of the lumen. Supportive intravenous fluid therapy was initiated for the azotemia and dehydration, and clopidogrel (1.8 mg/kg orally once daily) and pimobendan^4^ (0.23 mg/kg orally twice daily) were started. The previously prescribed dose of sildenafil was continued.

Overnight the patient's mentation and appetite improved. Serum biochemistry panel results the following morning revealed an unchanged blood urea nitrogen concentration (20.9 mmol/L), but an improved creatinine concentration (122.0 μmol/L) and serum total solid concentration (70.0 g/L). The presence of SEC was again observed on TTE. The patient was discharged with the instructions to continue the sildenafil and pimobendan until a recheck examination in 5 weeks. A 14-day supply of tramadol (4.4 mg/kg orally three times daily) was prescribed for pain. A 4-week supply of clopidogrel was sent home, so that the recheck examination would occur 1-week after discontinuation of the clopidogrel.

Approximately 5 weeks after discharge, the patient was presented for a recheck examination. The owner reported no concerns, instead noting that the dog seemed to have an improved energy level compared to pre-operatively. A serum biochemistry panel revealed that the azotemia was resolved (blood urea nitrogen 8.1 mmol/L and creatinine 123.8 μmol/L). Thoracic radiographs revealed a decrease in heart size with a vertebral heart size of 10.6 (Figure 1C). No SEC was noted on TTE (Figure 2C), and the internal diameter of the LV at end-systole normalized to body weight was normal at 1.1 (9). Fractional shortening was 40% (Figure 2F). The MPA had further decreased in size with a diameter of 1.6 cm. Pulmonic insufficiency maximal velocity was 1.8 m/s. The ACDO was still positioned appropriately, and there was no residual ductal flow (Figure 3C). All medications other than the sildenafil were discontinued. Based on the documented severity and chronicity of pulmonary arterial remodeling, PH was assumed to remain permanently. However, instructions to re-evaluate this with echocardiography in 6–8 months were given, so that if a resolution of PH was documented, the sildenafil could be discontinued. The patient has continued to do well as of the time of writing (6 months post-operatively).

## Discussion

Spontaneous echocardiographic contrast has been reported in a number of species including dogs, cats, and humans (12–15). It is typically diagnosed with TTE or transesophageal echocardiography, and it is described as a swirling signal of increased echogenicity within cardiac chambers or vessels. It is distinguished from excessive gain-induced white noise artifact due to its dynamic nature (12, 13). Multiple factors may lead to the aggregation of red blood cells and fibrinogen that result in SEC formation including blood stasis, hypercoagulability, and local endothelial injury (13–15). Spontaneous echocardiographic contrast is often associated with cardiac disease (12, 13, 15). In humans, SEC is commonly observed in the LA and/or left auricle and is associated with atrial fibrillation and diseases which cause LA enlargement such as mitral stenosis (12, 13). Additionally, SEC has been documented in the descending Ao and LV of humans with dilated cardiomyopathy (16–18). Regardless of underlying etiology, SEC is considered a risk factor for arterial thromboembolism in humans (12, 13, 17, 19). Identifying the presence of SEC is similarly concerning in cats. Cats with acquired cardiomyopathy have a significantly increased risk of increased mortality if SEC is present (15), and cats with hypertrophic cardiomyopathy had a hazard ratio of 9.4 for developing arterial thromboembolism in one study when SEC was present (20). Cats with an acquired cardiomyopathy most often develop SEC in their LA and/or left auricle in part due to chamber enlargement and secondary blood stasis (15, 21).

Spontaneous echocardiographic contrast is uncommonly reported in the dog (14, 22–24). One case series documented SEC in the LV (1/3) or all cardiac chambers (2/3) of three dogs with hyperfibrinogenemia (14). In this case series, dogs were diagnosed with infective mitral endocarditis, presumptive Evan's syndrome, or presumptive sepsis of unknown origin; however, these dogs had either normal cardiac size or mild cardiomegaly (14). Another dog with SEC was diagnosed with steroid-responsive meningitis-arteritis, pericardial effusion, a severely elevated C-reactive protein concentration, and a severely elevated cardiac troponin I concentration (23). This dog had SEC in the LV but had normal cardiac size and function of all chambers (23). Trauma associated with abdominal surgery has also been reported to cause SEC in the caudal vena cava in otherwise healthy dogs in a research setting (22). In these reports, the authors speculated that systemic inflammation caused the development of SEC (14, 22, 23). Diagnostic testing to investigate hypercoagulability such as thromboelastography or D-dimer concentrations may have been of benefit in this case, and the lack of these tests may be considered a limitation.

Dogs without systemic inflammation can also develop SEC. In one prospective study the effects of dexmedetomidine combined with butorphanol were evaluated in healthy dogs, and the authors observed that some dogs developed SEC after dexmedetomidine administration (24). All 14 dogs in that study had normal echocardiograms prior to dexmedetomidine administration. Fifty percent of dogs that received a higher dose of dexmedetomidine developed SEC, whereas 33% of dogs that received a lower dose of dexmedetomidine developed SEC (24). Administration of the alpha-2 agonist dexmedetomidine causes an increase in systemic afterload and secondary decreases in LV systolic function and heart rate (24, 25). The dog of our report experienced both a decrease in heart rate as well as an increase in systemic blood pressure acutely after ductal occlusion similar to what has been reported (26).

The dog in this report had clinically important PH, as assessed by the tricuspid regurgitant velocity, right heart remodeling, enlargement of the MPA, and the bidirectional ductal shunting. It is possible that the LV function was compromised secondary to the remodeling of the RV chamber and elevated RV pressure. This detrimental effect that an enlarged RV with elevated pressure has on LV function has been called the reverse Bernheim effect (27), and it occurs due to ventricular interdependence (28, 29). The dog of this report was 8-years-old, which may have contributed in part to LV dysfunction. Studies of older dogs with a PDA have documented the presence of LV dilation as well as systolic dysfunction (30, 31). It is possible that multiple components contributed to the LV systolic dysfunction and subsequent development of SEC in this dog: having clinically important PH, being older at diagnosis, and experiencing an acute increase in systemic afterload with ductal occlusion.

Clopidogrel was administered to the dog in this report at the time SEC was diagnosed. This decision was based on multiple factors. The presence of SEC was deemed to be a risk for systemic thromboembolism, based on reports in cats and humans (12, 13, 20). The dose of clopidogrel was chosen to provide prophylaxis against thromboembolism, with the intention of having minimal effect on the local thrombosis surrounding the ACDO (32). For this reason, a loading dose of clopidogrel was not given. Based on the lack of residual ductal flow as well as the unchanged position of the ACDO 5 weeks post-operatively, the formation of a thrombus and subsequent fibrosis around the ACDO was seemingly unaffected by this dose of clopidogrel. In one study evaluating healthy dogs, the authors describe the presence of SEC up to 60 min after dexmedetomidine administration (24). No dogs in that study had SEC prior to dexmedetomidine administration, and the SEC presumably resolved with metabolism and excretion of the sedative. However, whether the SEC resolved in all dogs and in what time frame this occurred in was not evaluated in that study. Based on the likelihood of persistence of PH and therefore of a reverse Bernheim effect, as well as the fact that some older dogs have persistent LV systolic dysfunction after PDA occlusion (31, 33), the authors believed that administering clopidogrel for thromboembolic prophylaxis was appropriate due to the potential for SEC persistence.

## Concluding Remarks

In conclusion, we describe the development of SEC in an older dog with a PDA and concurrent PH. A standard dose of clopidogrel was administered to prevent thromboembolism, and there were no clinical or biochemical derangements to suggest thromboembolism occurred. Thrombus formation around the ACDO was seemingly unaffected by this dose of clopidogrel, and the SEC was no longer present 5 weeks after diagnosis. To the authors' knowledge, this is the first report of SEC formation acutely after PDA occlusion in the dog. Dogs with a similar presentation of clinically important PH and an older age at time of PDA diagnosis and occlusion should be evaluated post-operatively for the presence of SEC. If SEC is diagnosed after PDA occlusion, it is possible that the dose of clopidogrel described here may allow simultaneous thromboembolism prophylaxis and the desired ACDO-associated thrombus formation.

## Data Availability Statement

The datasets generated for this study are available on request to the corresponding author.

## Ethics Statement

Ethical review and approval was not required for the animal study because this case describes clinical management of an individual animal. Written informed consent was obtained from the owners for the participation of their animals in this study.

## Author Contributions

RW and DN contributed to case management. RW and JR contributed to manuscript preparation. RW, DN, and JR contributed to manuscript editing.

### Conflict of Interest

The authors declare that the research was conducted in the absence of any commercial or financial relationships that could be construed as a potential conflict of interest.

## Footnotes

^1^Radiofocus® hydrophilic coated guidewire, Terumo Medical, Sumerset, NJ, USA.

^2^4Fr Flexor Check Flo catheter®, Cook® Medical, Bloomington, IN, USA.

^3^Amplatz® Canine Duct Occluder, Infiniti Medical, LLC™, Menlo Park, CA, USA.

^4^Vetmedin, Boehringer Ingelheim, Ridgefield, CT, USA.

## Supplementary Material

Supplemental Video 1 Right parasternal short axis video of the ventricles during the TTE exam performed ~24 h after ACDO deployment. Note the swirling columns of SEC within the lumen of the LV. ACDO, Amplatz® Canine Duct Occluder; LV, left ventricle; SEC, spontaneous echocardiographic contrast; TTE, transthoracic echocardiography.

## Abbreviations

ACDO: Amplatz® Canine Duct Occluder
Ao: Aorta
LA: Left atrium
LV: Left ventricle
MPA: Main pulmonary artery
PDA: Patent ductus arteriosus
PH: Pulmonary hypertension
RV: Right ventricle
SEC: Spontaneous echocardiographic contrast
TTE: Transthoracic echocardiography.
